# Bright Single-Photon Sources for the Telecommunication O-Band Based on an InAs Quantum Dot with (In)GaAs Asymmetric Barriers in a Photonic Nanoantenna

**DOI:** 10.3390/nano12091562

**Published:** 2022-05-05

**Authors:** Maxim Rakhlin, Grigorii Klimko, Sergey Sorokin, Marina Kulagina, Yurii Zadiranov, Dmitrii Kazanov, Tatiana Shubina, Sergey Ivanov, Alexey Toropov

**Affiliations:** Ioffe Institute, 194021 St. Petersburg, Russia; gklimko@mail.ru (G.K.); sorokin@beam.ioffe.ru (S.S.); marina.kulagina@mail.ioffe.ru (M.K.); zadiranov@mail.ioffe.ru (Y.Z.); kazanovdr@gmail.com (D.K.); shubina@beam.ioffe.ru (T.S.); ivan@beam.ioffe.ru (S.I.); toropov@beam.ioffe.ru (A.T.)

**Keywords:** quantum dots, single-photon emission, InAs, MBE, nanoantenna

## Abstract

We report on single-photon emitters for the telecommunication O-band (1260–1360 nm), which comprise an InAs/(In)GaAs quantum dot with asymmetric barriers, placed inside a semiconductor tapered nanocolumn acting as a photonic nanoantenna. The implemented design of the barriers provides a shift in the quantum dot radiation wavelength towards the O-band, while the nanoantenna collects the radiation and ensures its effective output. With non-resonant optical pumping, the average count rate of emitted single photons exceeds 10 MHz with the second-order correlation function $g^{\left(2\right)}$(0) = 0.18 at 8 K.

## 1. Introduction

Modern systems of optical quantum cryptography, providing high security during data transmission, imply the use of the sources of single photons, whose frequency coincides with one of the telecommunication spectral bands specific for the fiber optic networks [1,2,3,4]. The information transfer rate is determined by the emission intensity. The single-photon emitters with sufficiently high single-photon radiation rates are not currently available for use in the telecommunication systems, which hinders progress in this important area.

The most suitable systems for generating pure single photons on demand are single epitaxial quantum dots [5,6,7,8], possessing well-distinguishable quantum levels of excitons, trions, and biexcitons [9]. For the spectral range 900–950 nm, such sources based on single epitaxial InAs/GaAs quantum dots (QDs) placed in a columnar microresonator with Bragg reflectors or in a photonic crystal are currently commercially available [10,11]. However, despite the significant efforts of the world’s leading groups, attempts to master the telecommunication bands with the longer wavelengths have not been completely successful—in particular, due to a decrease in radiation intensity and ineffective extraction of light from single QDs in a planar microcavity [12]. Only T. Gao et al. demonstrated a quantum key distribution testbed using a plug-and-play telecom-wavelength single-photon source [13]. The best result to date is to achieve an end-to-end efficiency of 1.4% for the O-band with InGaAs/GaAs QDs located inside a circular Bragg grating (bull’s eye resonator) [14], although, as was shown for InAs/GaAs QDs emitting in the spectral range of 900–950 nm, this value can exceed 50% [15].

There are several ways to shift the QD radiation wavelength towards longer wavelengths. The first one is an increase in the QD size. The disadvantage of this method is the difficulty of realizing a sufficiently low surface density of the QDs fabricated with a conventional Stranski–Krastanow method of self-organized growth [16]. In addition, for large QDs, a weak dependence of the radiation wavelength on their shape and size is observed. As a result, the emission spectra of different dots overlap with each other, and separation of the radiation from a single QD becomes impossible. Another method involves the use of a ternary InGaAs alloy as a matrix instead of the GaAs binary compound [17]. In this case, a metamorphic buffer layer is often introduced to align the lattice constant of the GaAs substrate and the InGaAs matrix. This approach leads to a decrease in the lattice mismatch between the materials of the matrix and QDs [18], which undesirably reduces the stress, which is the driving force for the formation of QDs. As a result, the QDs are characterized by irreproducible size, shape, and lateral density.

In our work, we form the InAs QDs by molecular beam epitaxy (MBE) directly on the GaAs buffer layer, which ensures the highest possible stress level. The increase in the radiation wavelength is achieved due to the asymmetry of the barriers surrounding the QDs. Namely, the upper barrier layer is made of an InGaAs ternary alloy with a band gap smaller than that of GaAs. As a result, the size and density of QDs are basically preserved, while the radiation wavelength is significantly enlarged. This approach was first proposed by Alloing et al. [19]; then, it was applied for the entangled photon pair generation [20] and observing the Mollow triplet in the Rabi regime [21]. However, to the best of our knowledge, bright single-photon radiation using asymmetric barriers has not been obtained so far. One of the most promising ways to enhance the luminescence intensity is the use of plasmonic nanostructures that provide amplification of the electromagnetic field on the nanoscale [22,23]. However, in this work, we will focus on semiconductor photonic nanoantennas, which is primarily a way to maximize the efficiency of radiation extraction. They are single-mode columnar (“cylindrical”) waveguides with the diameter continuously varying along its axis. Such a design is characterized by the high efficiency of photon extraction in a wide spectral range of several tens of nanometers [24,25,26,27]. The realized efficiency of emission extraction allowed us to implement the sources with the average emission rate exceeding 10 MHz at 8 K.

## 2. Sample Fabrication and Experimental Techniques

The InAs/(In)GaAs QD structure was grown on a (001)-oriented n${}^{+}$-GaAs substrate using an MBE setup (SemiTEq) equipped with two Ga and In hot-lip effusion cells, as well as an As valve cracking cell. The sample consisted of a 200-nm-thick GaAs buffer layer grown at a substrate temperature T_s_ = 590 °C to provide a smooth surface for the subsequent deposition of InAs QDs. Immediately after the growth of the GaAs buffer layer, the substrate temperature was decreased to T${}_{s}=480\;$°C. The QD deposition temperature was controlled with reflection high-energy electron diffraction, indicating the transition of the GaAs surface reconstruction from (2 × 4)As to c(4 × 4)As under As flux. Typically, most QDs emitting at 1.3 μm possess lateral sizes 40–60 nm and a height 9–12 nm [12].

The QDs were formed in the Stranski–Krastanow mode by the deposition of 2 monolayer (ML) InAs at a low growth rate of 0.002 ML/s. After closing the In shutter, the growth surface was exposed to As flux for 60 s. Then, the substrate temperature was rapidly lowered down to 460 °C to suppress the mutual diffusion of In and Ga atoms during coverage of the InAs inserts. The formed InAs QDs were covered by 5-nm-thick In${}_{0.18}$Ga${}_{0.82}$As followed by a 5-nm-thick GaAs layer with a growth rate of ∼1.6 nm/min. The rest of the 2.5-μm-thick GaAs cap layer was grown with an average growth rate of 10 nm/min at the substrate temperature of 520 °C.

**Figure 1:**
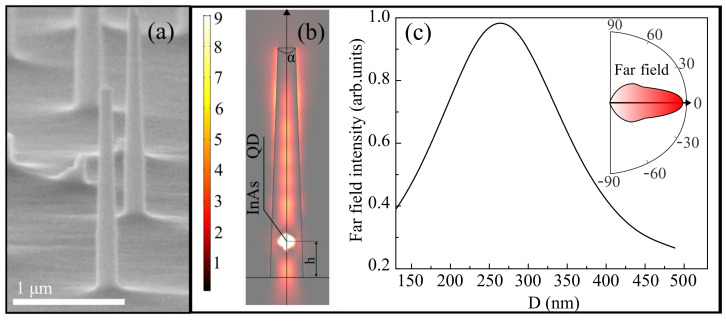
(**a**) Scanning electron microscopy image of the photonic nanoantennas. (**b**) Typical near-field distribution of electromagnetic energy in a photonic nanoantenna. (**c**) Far-field radiation intensity of nanoantenna, calculated for different diameters of the nanoantenna’s base, normalized to maximum intensity. Inset shows the typical far-field angular distribution of the intensity.

Photonic nanoantennas in the form of columns tapering to the tops were formed by etching in a combined plasma of a capacitive and inductive discharge in a plasma–chemical etching setup, STE ICP 200e (SemiTEq). BCl_3_ was used as a reactive gas, and Ar as an auxiliary gas. Sapphire nanospheres were used as masks to protect the material from etching. As a result, columnar structures with a variable cross-section were obtained, approximately 2.8 μm in height, 280–320 nm in diameter at the base, and 170–200 nm at the apex (Figure 1a). The density of nanoantennas located at random positions, determined by the number of nanospheres per unit area, was low enough to provide the possibility to detect the emission of single nanoantennas containing a small number of emitting QDs (or even only a single QD) in the basement. The nanoantenna with the most intense excitonic emission line of a single QD was selected for further investigation.

Micro-photoluminescence (μ-PL) spectroscopy was used to investigate the luminescence properties of the sample with nanoantennas. The μ-PL spectra were measured at a temperature 8 K using a helium-flow cryostat, ST-500-Attocube (Janis). The sample was adjusted using a three-coordinate piezo-driver with an accuracy of approximately 20 nm. These nanopositioners were located directly in the cold zone of the cryostat, which allowed us to achieve high mechanical stability and vibration isolation during the measurement. PL excitation was carried out by focusing cw laser radiation (405 nm) on a sample with a minimum spot size of 3–5 μm and a power density of less than 4 W/cm^2^. Focusing was carried out with an objective (Mitutoyo plan apochromat with 50× magnification, NA = 0.42), which was also used to collect the signal. The PL radiation transmitted through the objective was focused by a triplet achromatic lens in the plane of a mirror with a calibrated aperture (pinhole). The PL radiation passed through the aperture was focused into the entrance slit of an SP-2500 spectrometer (Princeton Instruments) with grating 1200 g/mm, providing a spectral resolution ∼70 μeV. We additionally used a bandpass interference filter in order to block scattered laser radiation from the sample and optical elements. A cooled PyLoN-IR CCD (Princeton Instruments) was used as a PL detector.

To measure the time-resolved PL (TRPL) spectra, we used excitation by a picosecond pulsed semiconductor laser PILAS 405 nm (Advanced Laser Systems) with pulse repetition frequency 100 MHz and power density ∼4 W/cm^2^. Superconducting single-photon detectors (Scontel) with a time resolution of approximately 40 ps were used as a detector for the TRPL. To register photons, a computer with an installed time-correlated single-photon counting system, SPC-130 (Becker & Hickl), was used.

## 3. Single-Photon Emitters with Single-Mode Nanoantenna

To verify our approach, we performed calculations of both near- and far-field intensities as well as mode analysis of the nanocolumns, using a finite-difference mode solver in Comsol Multiphysics. Modal analysis of the waveguide modes in the nanoantenna was performed in 2D regime in approximation of the infinite cylinder in the frequency domain that was located in the air and surrounded by a perfectly matched cylindrical layer. The mesh in the calculation was chosen fine (wavelength of the discussed frequency divided by ∼10). We obtained a propagation constant and an effective refractive index close to the refractive index of the structure for the pair of lowest modes near the frequency under discussion [28]. This approach allowed us to take a first close look at the electromagnetic wave inside the nanocolumn. In order to find the intensity of the electromagnetic field inside and outside the nanoantenna, we performed 3D finite difference frequency domain calculations [27]. The contribution to the PL intensity of QDs polarized perpendicular to the nanoantenna axis is much higher as compared to those polarized in the parallel direction, since QDs can be approximated by a dipole lying in the growth plane and, accordingly, emitting perpendicularly. Thus, to simulate the QD, we used the in-plane point-like dipole source placed at the center of the nanoantenna and oriented perpendicular to the column axis at a specific height h = 300 nm. We have found that this particular height makes it possible to increase the PL intensity due to the coincidence of the position of the QD with the antinode of the electric field in the *z* direction. The typical near-field electromagnetic energy distribution in the nanoantenna is depicted in Figure 1b. The mode calculations have shown that at the wavelengths of the O-band, the GaAs nanoantennas with the diameter lower than 280 nm at the basal part support only a single fundamental HE_11_ waveguide mode (that corresponds to the diameter of the fabricated nanoantenna; see Figure 1a). The following modes with vanishing field in the center of the nanoantenna and higher energy (TE_01_ and TM_01_) have a cut-off at a diameter of ∼285 nm. The next mode with even higher energy is HE_21_; it has a non-zero field in the center of the nanoantenna and corresponds to the diameter of the base ∼430 nm. Such a design of nanoantennas also provides strong confinement of the fundamental mode and coupling to the QD emission, while nanoantennas with a larger radius can support multiple modes [29]. Provided that the upper part of the nanocolumn is quite thin, the HE_11_ mode effectively leaks outside and forms a Gaussian-like plane wavefront.

The transverse confinement of the mode strongly depends on the diameter of the nanoantenna and should be optimized [7,28,30]. Small-diameter nanoantennas have less coupling of the QDs with the HE_11_ mode, since part of the wave is located outside the nanoantenna and interacts more weakly with the emitter. A too large diameter reduces the lateral confinement of light and decreases the coupling of the emitter and the fundamental mode. We calculated the far-field distribution using the built-in Stratton–Chu transform from the calculated near field. Figure 1c shows the radiation intensity of the nanoantenna in the far field for different diameters of the base, normalized to maximum intensity. The calculations were carried out at a tapered angle α=3.5° (dashed line in Figure 1b) that corresponds to a high radiation extraction efficiency from the photonic nanoantenna [24,25]. The highest radiation intensity in the far field is observed at a nanoantenna diameter ∼260 nm. The dependence of the far-field intensity on the diameter is not symmetric due to the appearance of higher-order modes with the non-zero field in the center of the nanoantenna with base diameter more than 430 nm. The inset of Figure 1c shows the typical far-field angular intensity distribution. The lens with NA =0.42, used in our experiment, collects 52% of the light emitted from a photonic nanoantenna. An increase in the collection efficiency up to 85% can be achieved using a lens with NA =0.7. We have also run the far-field calculations for layered structures without any nanoantennas and obtained more than one order lower intensity. Thus, the use of a photonic nanoantenna is a key factor providing an increase in the collected far-field radiation intensity. We emphasize that this is an alternative method of single-photon radiation enhancement compared to others, such as plasmonic effects in nanostructures [31,32,33].

## 4. Measurements and Quantum Statistics

**Figure 2:**
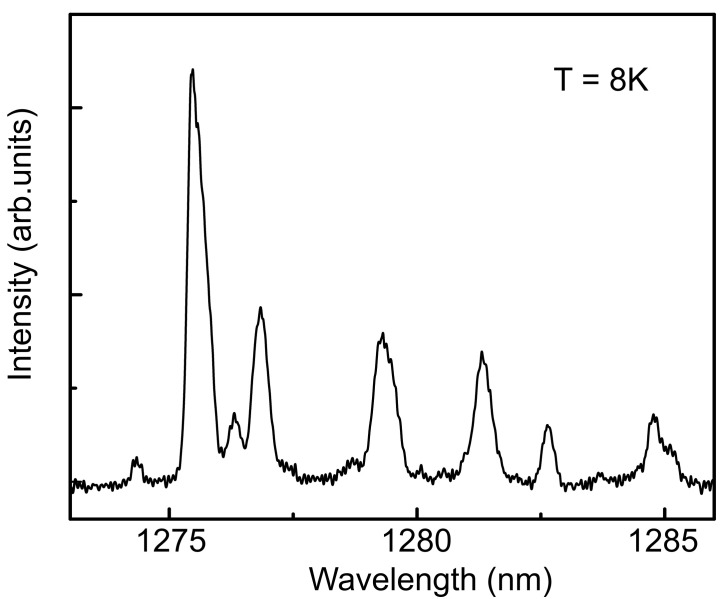
A representative μ-PL spectrum measured in a photonic nanoantenna with InAs QDs at 8 K.

**Figure 3:**
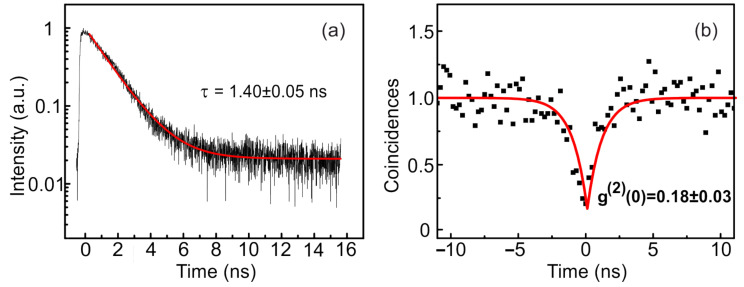
(**a**) Characteristic decay curve of the radiation from the QD in the photonic nanoantenna with the characteristic decay time of 1.4 ns. (**b**) Normalized second-order correlation function $g^{\left(2\right)}$ of single-photon emission, measured at 8 K for a spectrally filtered single excitonic line. The obtained value of $g^{\left(2\right)}$(0) is 0.18. The autocorrelation function and decay curve were measured at the most intensive excitonic peak in Figure 2.

A typical μ-PL spectrum measured at 8 K in a single photonic structure includes separate single lines associated with the emission of a small number of individual QDs located inside the nanoantenna (Figure 2). These lines can be assigned to the emission of neutral excitons and electron–hole complexes: biexcitons and trions. The spectrometer was used to isolate one selected exciton line from others. The decay dynamics of the most intense peak in the μ-PL spectrum is shown in Figure 3a. The simplest single-exponential fitting corresponds to a characteristic decay time of 1.40 ± 0.05 ns, which is typical for the recombination of excitons in InAs QDs at the telecommunication wavelength [12].

The second-order correlation function $g^{\left(2\right)}\left(\tau \right)$ obtained for this excitonic line is shown in Figure 3b. To obtain $g^{\left(2\right)}\left(\tau \right)$ at the appropriate scale, it is necessary to normalize the measured coincidence statistics [34,35]. The function is normalized according to the formula:

(1) $$\begin{array}{c} C_{N}\left(t\right)=c\left(t\right)/\left(N{}_{1}N_{2}T\omega \right), \end{array}$$

where c(t) is a raw coincidence number, $N_{1,2}$ are the average numbers of photons recorded by detectors per second, ω is the width of time bin (58.6 ps), and *T* is the total acquisition time (∼300 s).

The second-order correlation function can be approximated using the equation:

(2) $$\begin{array}{c} g^{\left(2\right)}\left(\tau \right)=a-b*e^{-\tau /c}, \end{array}$$

where *a*, *b*, and *c* are fitting parameters. The value of the parameter $c_{1}=1.3$ ns gives an estimate for the dip width of the correlation function at zero delay, which directly correlates with the radiative lifetime of the exciton. The average number of photons recorded by one detector per second for this structure was ∼1.4 × 10${}^{4}$ for 8 K, which, taking into account the instrument function of the measuring equipment (the efficiency of the radiation input into a single-mode fiber is approximately 2.5%, the losses in the spectrometer are approximately 4 times, and the losses in the optical scheme are approximately 2.5 times), corresponds to the intensity of single-photon emission on the first lens ∼10 MHz. The approximation of the experimental data using Equation (2) gave the value of the correlation function $g^{\left(2\right)}\left(0\right)=0.18\pm 0.03$, which is clear evidence of the single-photon nature of the emission [36,37].

## 5. Conclusions

To summarize, we have proposed a method for fabricating single-photon emitters based on InAs QDs emitting in the telecommunication O-range by using asymmetric barriers: the bottom one is made from binary GaAs, while the top is made from ternary InGaAs alloy. The obtained QD density has made it possible to study the single emission lines of individual QDs confined within the photonic nanoantenna with a base diameter of 280–320 nm and a height of 2.8 μm. The structures demonstrated single-photon emission with an average count rate of more than 10 MHz at 8 K. The value of the second-order correlation function $g^{\left(2\right)}\left(0\right)$ was approximately 0.18 at the wavelength of 1276 nm. A further increase in the purity of the single-photon emission can be achieved by using resonant optical pumping [38], while creating a single-photon source with a higher emission intensity requires even better control of the photonic nanoantenna shape during post-growth processing and the application of advanced cryolithography allowing precise matching of the spatial positions of the QD and nanoantenna. The achieved characteristics are good prerequisites for the application of the proposed single-photon emitter design in fiber-optic cryptography systems intended for the O-band.

## Data Availability

The data that support the findings of this study are available within the article.
